# Supplementary material for: An open-source framework for large-scale, flexible evaluation of biomedical text mining systems
Source: J Biomed Discov Collab. 2008 Jan 29;3:1. doi: 10.1186/1747-5333-3-1 (PMC2276192; doi:10.1186/1747-5333-3-1)
Supplement: Additional file 1 — Evaluation platform source code. The additional file contains the software evaluation framework discussed in this paper. The most current release can be downloaded from SourceForge.net [1]. See the accompanying README file for instructions on installation and use. Also included in the distribution is a collection of UIMA wrappers for some commonly used BioNLP tools and annotated corpora. [file 1747-5333-3-1-S1.zip › bionlp_uima_repository-1.0.1/docs/api/src-html/edu/uchsc/ccp/uima/cc/summary/comparison/ComparisonGroup.html]

```
001    package edu.uchsc.ccp.uima.cc.summary.comparison;
002    
003    import java.io.PrintStream;
004    import java.util.ArrayList;
005    import java.util.List;
006    
007    public class ComparisonGroup {
008    
009        private boolean isGoldStandard = false;
010    
011        private String description;
012    
013        private List<AnnotationGroupID> annotationGroupMembersList;
014    
015        public ComparisonGroup() {
016            this.isGoldStandard = false;
017            this.description = null;
018            this.annotationGroupMembersList = new ArrayList<AnnotationGroupID>();
019        }
020    
021        /* ComparisonGroup is valid as long as it has at least one AnnotationGroup member associated with it */
022        public boolean isValid() {
023            if (annotationGroupMembersList.size() != 0) {
024                return true;
025            } else {
026                return false;
027            }
028        }
029    
030        public List<AnnotationGroupID> getAnnotationGroupList() {
031            return annotationGroupMembersList;
032        }
033    
034        public void setAnnotationGroupList(List<AnnotationGroupID> annotationGroupMembersList) {
035            this.annotationGroupMembersList = annotationGroupMembersList;
036        }
037    
038        public void addAnnotationGroup(AnnotationGroupID group) {
039            this.annotationGroupMembersList.add(group);
040        }
041    
042        public String getDescription() {
043            return description;
044        }
045    
046        public void setDescription(String description) {
047            this.description = description;
048        }
049    
050        public boolean isGoldStandard() {
051            return isGoldStandard;
052        }
053    
054        public void setGoldStandard(boolean isGoldStandard) {
055            this.isGoldStandard = isGoldStandard;
056        }
057        
058        public void print(PrintStream ps) {
059            String membersStr = "";
060            for (AnnotationGroupID  groupID: this.annotationGroupMembersList) {
061                membersStr += (groupID + ", ");
062            }
063            membersStr = membersStr.substring(0, membersStr.lastIndexOf(","));
064            ps.println("ComparisonGroup: isGold: " + this.isGoldStandard + " Description: " + this.description + " Members: " + membersStr);
065        }
066    
067    }
```
